# Duodenal Amyloidosis Masquerading as Iron Deficiency Anemia

**DOI:** 10.7759/cureus.725

**Published:** 2016-08-05

**Authors:** Faisal Inayat, Abu Hurairah

**Affiliations:** 1 Department of Medicine, New York-Presbyterian Hospital, Weill Cornell Medical College; 2 Division of Gastroenterology, Department of Medicine, SUNY Downstate Medical Center

**Keywords:** gastrointestinal amyloidosis, iron deficiency anemia

## Abstract

The present study is a unique illustration of duodenal amyloidosis initially manifesting with iron deficiency anemia. It underscores the importance of clinical suspicion of amyloidosis while performing upper gastrointestinal endoscopy with a biopsy to establish the definite diagnosis in patients with unexplained iron deficiency anemia.

## Introduction

Amyloidosis results from extracellular deposits of fibrillar proteins in various organs. There are multiple types of amyloidosis defined by different precursor proteins. The classifications of amyloidosis include primary, secondary, hemodialysis-related, hereditary, senile, and localized.

Primary Amyloid Light-chain (AL) amyloidosis is a multisystem disease of unknown etiology characterized by deposition of amyloid fibrils composed of variable portions of the monoclonal light chains. AL Amyloidosis has been associated with neoplastic diseases such as multiple myelomas and B-cell lymphoma [1].

Reactive systemic Amyloid A (AA) amyloidosis, formerly known as secondary amyloidosis, is composed of a non-immunoglobulin, amyloid A. It usually complicates various chronic infections. In non-HIV-infected persons, AA amyloid commonly affects the spleen, lymph nodes, kidney and liver [2]. However, duodenal AA amyloidosis with complete sparing of the colon, initially presenting with iron deficiency anemia, is a rare clinical event.

## Case presentation

A 79-year-old male with a history of diabetes mellitus presented to the State University of New York (SUNY) Downstate Medical Center Gastroenterology Department with an unexplained iron deficiency anemia. His initial physical examination revealed a body mass index (BMI) of 20.7 (18.5-24.9). The abdominal exam showed a slightly distended abdomen with normal intestinal peristaltic sounds.

Chest radiography, electrocardiogram, and echocardiography showed no abnormalities. Computed tomography abdomen and ultrasonography were unremarkable. Barium studies showed malrotation of the small bowel. The proximal small bowel loops appeared in the right abdomen. However, there was no clinical evidence of intestinal obstruction. Esophagogastroduodenoscopy (EGD) showed duodenitis, with no *Helicobacter pylori*, whereas colonoscopy revealed no abnormalities.

Initial laboratory studies revealed iron deficiency anemia with hemoglobin 6.5 mg/dL (normal, 13.5-17.5 mg/dL), serum ferritin 12 ng/mL (normal, 24-336 ng/mL), mean corpuscular volume (MCV) 72 fL/red cell (normal, 80-96 fL/red cell), and total iron binding capacity (TIBC) 512 mcg/dL (240-450 mcg/dL). In addition, a hypoalbuminemia of 2.0 mg/dL (normal, 3.5-5.5 mg/dL) was observed. Urinary findings showed no abnormalities. The patient had normal complement, Thyroid-stimulating hormone (TSH), C-reactive protein (CRP), and serum creatinine levels with negative Coombs test. Liver function tests were within normal limits with a negative hepatitis profile.

**Figure 1:**
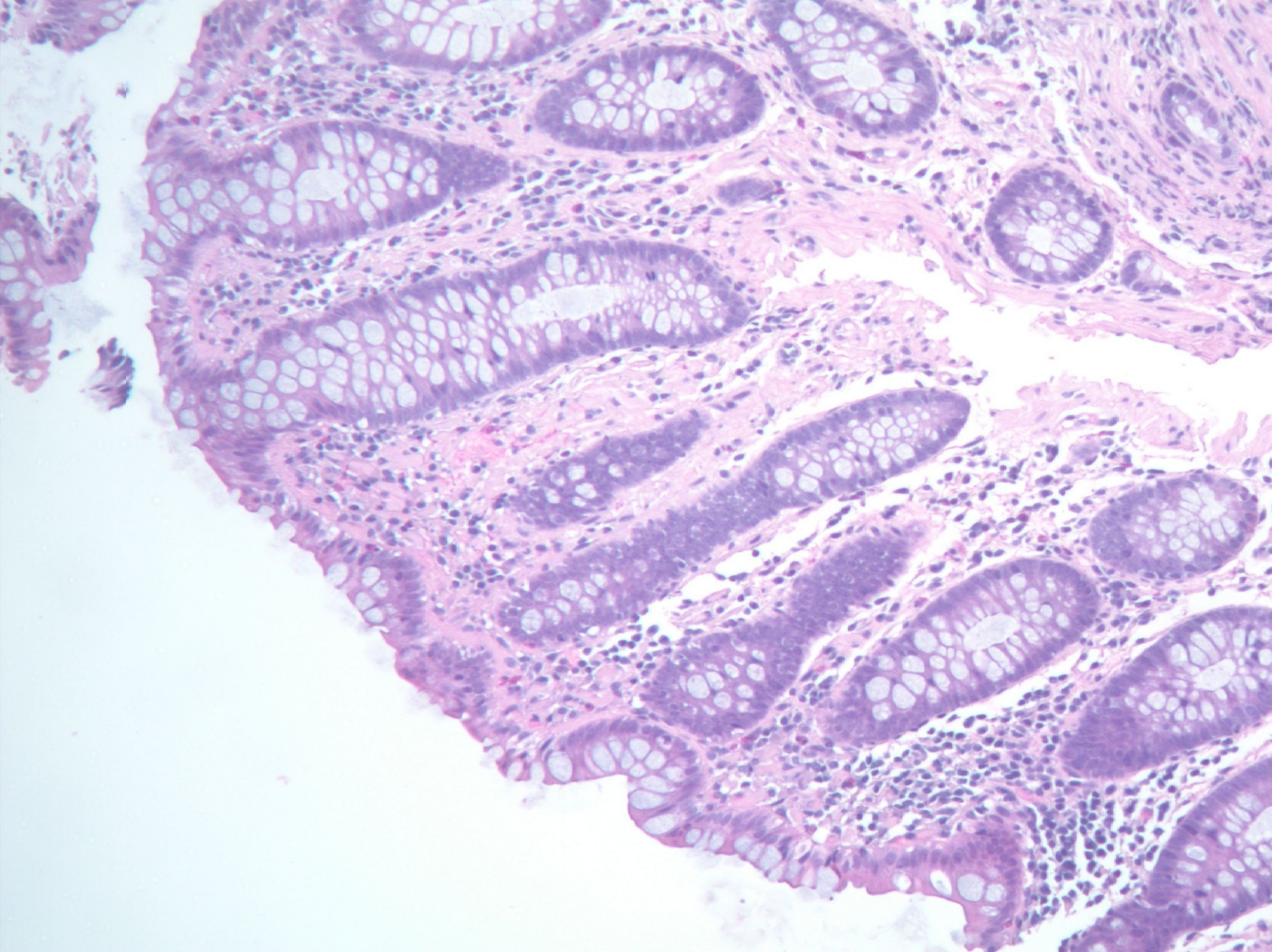
Duodenal Biopsy Photomicrograph reveals mucosal biopsy showing glassy pink material in the lamina propria.

Histopathologic analysis of small bowel mucosal biopsy specimens revealed glassy pink material in the lamina propria. (see Figure 1)

**Figure 2:**
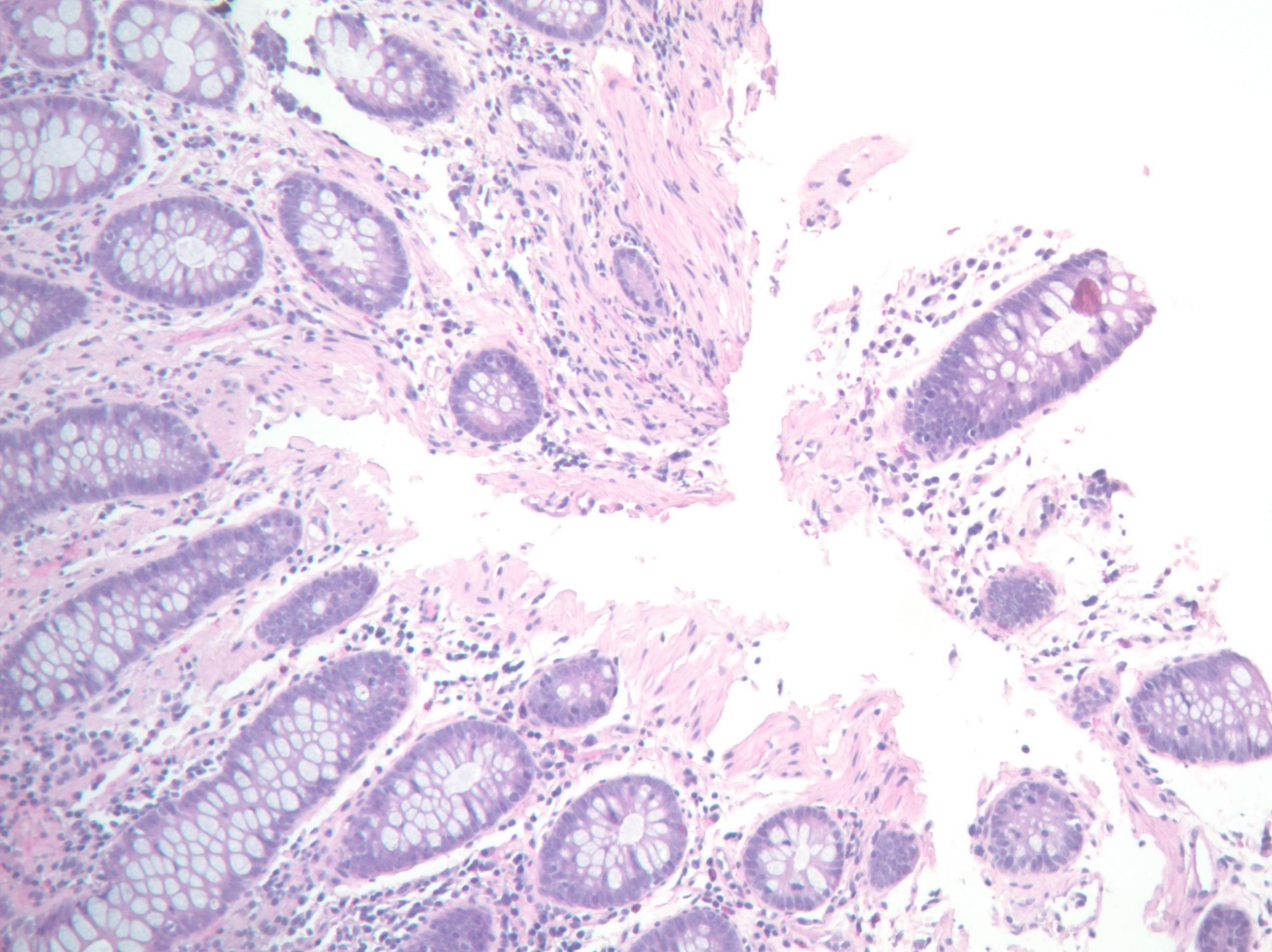
Intact Mucosal Biopsy of the Duodenum Photomicrograph reveals a glassy pink material in the lamina propria with mild to moderate chronic inflammatory infiltrate predominantly composed of lymphocytes.

Furthermore, chronic inflammatory infiltrates predominantly composed of lymphocytes were identified in the duodenal mucosa. (see Figure 2)

**Figure 3:**
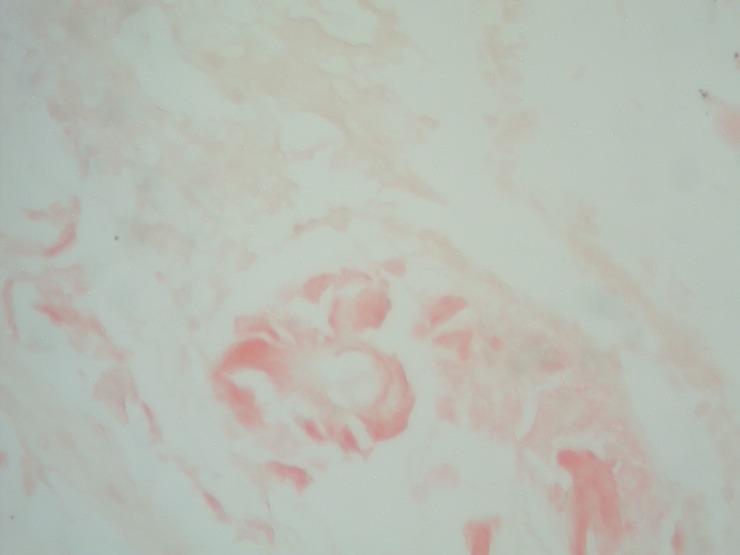
Duodenal Biopsy Specimen Stained with Congo Red Dye Photomicrograph showing positive Congo Red staining of the glassy pink material in the lamina propria from a duodenal biopsy confirming the amyloidosis type A deposition.

Congo Red staining demonstrated the amyloid A deposits in the duodenal biopsy specimens. (see Figure 3)

These observations confirmed the diagnosis of duodenal AA amyloidosis.

Colon biopsy showed no amyloid deposition. Bone marrow biopsy revealed normocellular marrow without excess blasts or dysplastic changes ruling out the malignancy. Furthermore, workup for primary AL amyloidosis including serum protein electrophoresis (SPEP) and serum free light chains (FLC) assay came out negative. Based on the findings described above, we diagnosed iron deficiency anemia as an initial symptom of duodenal amyloidosis. Additionally, the patient did not display any clinical signs of heart failure. Renal function tests were also within normal limits.

The patient was administered 1.2 mg/day with iron supplementation. One month follow-up laboratory studies showed improvement in hemoglobin, and her subsequent serum ferritin level was 53 ng/mL (normal, 24-336 ng/mL). The patient has been off iron supplementation for six months now and continued to do well. 

## Discussion

The gastrointestinal amyloidosis may pose a diagnostic challenge to clinicians. Despite the fact that diagnosis of amyloidosis requires a histologic demonstration of amyloid deposits; gastrointestinal amyloidosis, with the biopsy-proven disease, is rare. Cowan AJ et al. retrospectively reviewed 2,334 patients with all types of amyloidosis presented in a period of over 13 years [2]. Only 76 patients (3.2%) had biopsy-proven amyloid involvement of the gastrointestinal tract.

Endoscopic features of the amyloidosis in the upper gastrointestinal tract are variable, ranging from subtle erosive changes, mucosal friability and granularity to polypoidal protrusions [2]. The patient in the present report was ultimately found to have diffused granular erosive changes at the proximal duodenum at upper gastrointestinal endoscopy with histological verification of AA amyloid deposition in the absence of colonic disease on histologic assessment. The present report represents only the second reported case where the disease completely spared the colon [3].

Gastrointestinal hemorrhage occurs as a presenting symptom in 25-45% of patients with gastrointestinal amyloidosis and may be caused by ischemia or infarction, by ulceration or an infiltrated lesion, or from generalized oozing without a particular source [4]. However, the present patient is unique as he had no amyloid-related gastrointestinal symptoms and presented only with iron deficiency anemia. Despite extensive investigations including repeat colonoscopy with biopsy, capsule endoscopy, stool studies, urine 5-hydroxyindoleacetic acid (5-HIAA) levels, fecal fat, hydrogen breath tests (X2), serum and urine protein electrophoresis (SPEP, UPEP); no apparent cause for blood loss was evident. Therefore, in the present patient, amyloid infiltration of duodenum might have contributed to the iron deficiency anemia keeping in mind the fact that the predominant site of iron absorption is duodenum. Also, duodenitis may have contributed to the presentation. 

AA amyloidosis is associated with infectious, inflammatory, or less commonly, neoplastic disorders [5]. Rheumatoid arthritis is the most common cause of AA amyloidosis [5]. Other inflammatory disorders such as Crohn’s disease, ankylosing spondylitis, primary biliary cirrhosis, familial Mediterranean fever, and systemic lupus erythematosus are also associated with this disorder [5]. The present patient did not have a history of chronic inflammatory or infectious conditions. However, his past medical history was remarkable for diabetes mellitus. 

Autoimmune diseases or type 2 diabetes mellitus may be potential risk factors for AA amyloidosis. In tissues of patients with autoimmune diseases, the deposition of antibody-antigen immune complexes causes chronic inflammation [6]. Furthermore, in the blood of people with diabetes mellitus, serum amyloid A (SAA) protein levels are relatively high compared to those of healthy people [7]. SAA is the acute phase reactant, which can increase a thousand-fold during inflammation and remains persistently elevated until the inflammation resolves. However, clinical evidence suggesting the causal relationship between diabetes mellitus and development of amyloidosis AA is sparse.

## Conclusions

Iron deficiency anemia as the first presentation of underlying gastrointestinal amyloidosis is unusual but clinically significant. Although a rare manifestation of amyloidosis, staining for amyloid should be considered in patients undergoing gastrointestinal biopsy who have unexplained iron deficiency anemia.
